# Laparoscopic Evaluation of Traumatic Pneumoperitoneum Without Hollow Viscus Injury: A Case Series

**DOI:** 10.7759/cureus.94214

**Published:** 2025-10-09

**Authors:** Hideo Kidogawa, Takeshi Konno, Takatomo Yamayoshi, Masao Inoue, Kohji Okamoto

**Affiliations:** 1 Department of Surgery, Kitakyushu City Yahata Hospital, Kitakyushu, JPN

**Keywords:** blunt abdominal trauma, diagnostic laparoscopy, negative laparotomy, non-surgical pneumoperitoneum, pneumoperitoneum

## Abstract

The presence of free pneumoperitoneum (free intraperitoneal air) after blunt trauma is generally considered indicative of a hollow viscus perforation, often necessitating emergency surgery. This finding presents a major diagnostic challenge in multiply injured patients. Here, we present three cases of traumatic pneumoperitoneum in multiply injured patients without visceral perforation. All patients had sustained high-energy trauma resulting in severe associated injuries, including thoracic trauma and multiple fractures. Diagnostic laparoscopy was utilized to evaluate the abdomen in each case and successfully ruled out intra-abdominal injuries, thus avoiding the morbidity of an unnecessary laparotomy. Our experience suggests that diagnostic laparoscopy can be a safe and effective tool for the evaluation of hemodynamically stable, multiply injured patients with traumatic pneumoperitoneum of an unclear etiology.

## Introduction

Blunt abdominal trauma (an injury to the abdomen caused by impact rather than a penetrating object) is a leading cause of morbidity and mortality in multiply injured patients (those with significant injuries to multiple body regions or systems), with hollow viscus injury (HVI) being a particularly life-threatening condition requiring prompt diagnosis and surgical intervention. The presence of pneumoperitoneum on CT has long been regarded as a cardinal sign of HVI. However, this diagnostic algorithm is increasingly challenged by the recognition that pneumoperitoneum can arise from non-surgical causes [1,2], and that its presence on CT can be an unreliable predictor of intra-abdominal injury in blunt trauma [3].

This creates a significant clinical dilemma. The rate of negative laparotomies in trauma patients remains substantial, and these procedures are associated with significant morbidity, including increased complications and prolonged hospital stays [4]. The decision-making process is further complicated in hemodynamically stable (presenting with normal blood pressure and not in shock), multiply injured patients, where distracting injuries and altered mental status can render physical examinations unreliable. Consequently, surgeons must balance the risk of a morbid negative laparotomy against the potentially fatal consequences of a missed HVI.

In this context, diagnostic laparoscopy has emerged as a valuable, minimally invasive tool for evaluating stable trauma patients with equivocal abdominal findings. It allows for direct visualization of the peritoneal cavity, enabling surgeons to definitively rule out visceral injury and thereby avoid the risks of a full laparotomy. This case series presents our experience with three patients who presented with traumatic pneumoperitoneum without visceral injury, aiming to highlight the pivotal role of diagnostic laparoscopy in navigating this diagnostic gray zone and guiding appropriate patient management.

## Case presentation

Surgical approach

At our institution, our standard protocol for hemodynamically stable trauma patients, including both blunt and penetrating injuries, is to proceed with laparoscopic surgery. The initial exploration is performed using a single-incision laparoscopic approach. If an injury is identified, repair is attempted laparoscopically. In cases of bowel or mesenteric injury, the injured segment is exteriorized through the single-port incision for repair.

Case 1

**Figure 1:**
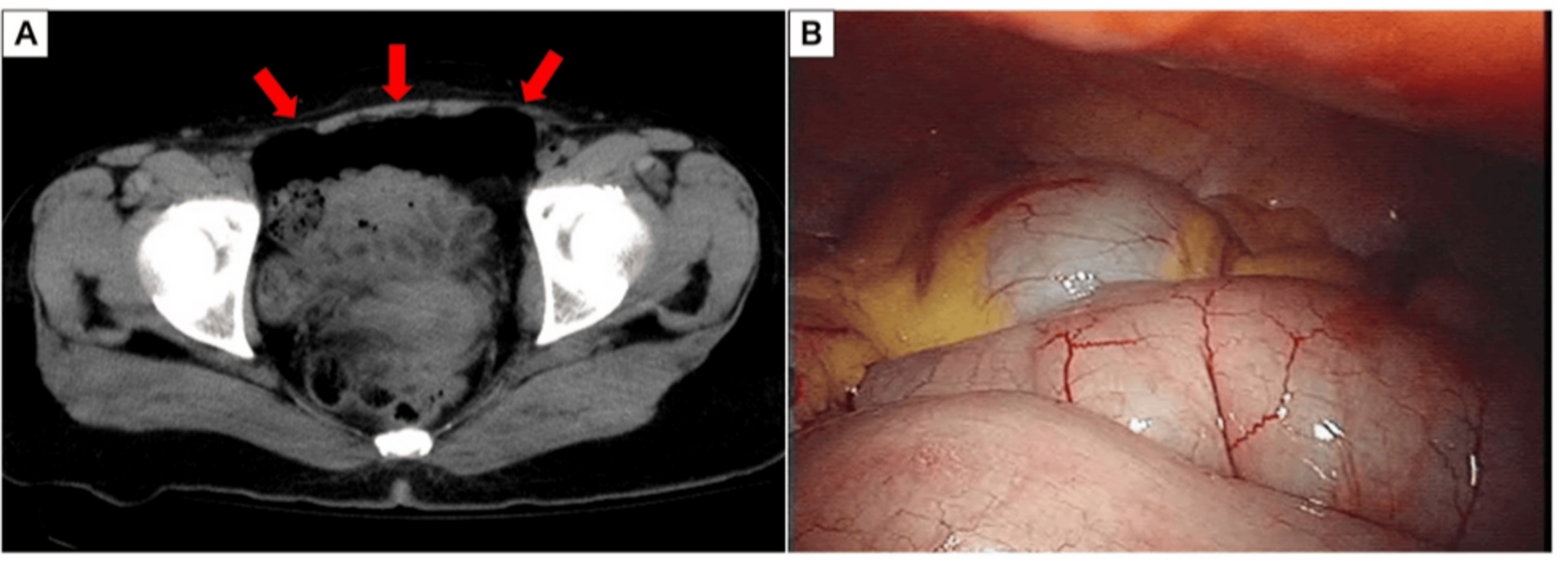
Imaging and intraoperative findings of Case 1. (A) Axial CT image of the lower abdomen showing free intraperitoneal gas (red arrows). (B) Laparoscopic view after a thorough exploration of the peritoneal cavity. No evidence of hollow viscus perforation or contamination was identified.

A 48-year-old female with a history of schizophrenia was transported to our emergency department after jumping from the fifth floor of her apartment building, landing on a light motor vehicle. On admission, her systolic blood pressure was 90 mmHg. She presented with an altered mental status, and a reliable physical examination of the abdomen could not be performed. Laboratory findings were unremarkable, with a white blood cell (WBC) count of 8,640/μL and C-reactive protein (CRP) of 0.0 mg/dL. A whole-body CT scan revealed multiple injuries, including bilateral hemopneumothorax, pelvic fracture, thoracolumbar fractures, and free intraperitoneal air (Figure 1a). Bilateral chest tubes were placed in the emergency department upon diagnosis of the hemopneumothorax. Given the free air, a diagnostic laparoscopy was performed. Laparoscopic exploration showed no evidence of gastrointestinal injury but confirmed a mild retroperitoneal hematoma on the lateral side of the duodenum (Figure 1b). Due to the possibility of a duodenal injury, a mini-laparotomy with a Kocher maneuver was performed to definitively exclude a perforation. No duodenal injury was identified, and the hematoma was therefore managed conservatively. The patient’s thoracolumbar injuries were determined to be compression fractures, not a Chance-type fracture. As a precaution against missed injuries, two drains were placed: a Blead drain in the pouch of Douglas and a Penrose drain in the subhepatic space via the right flank. Postoperatively, she was monitored in the intensive care unit. The abdominal drains were removed on the third postoperative day. The chest drain was removed on the ninth postoperative day, and she was transferred to the neurosurgery department for further surgical management on the tenth postoperative day.

Case 2

**Figure 2:**
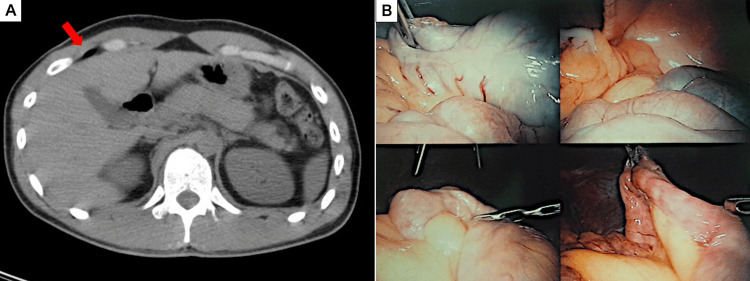
Imaging and intraoperative findings of Case 2. (A) Axial CT image revealing free air on the ventral side of the liver (red arrows). (B) A systematic laparoscopic survey of all abdominal quadrants confirming the absence of visceral injury or hemorrhage.

A 27-year-old male was crushed by a 700-kg panel and transported to our emergency department. He was hemodynamically stable on arrival. He presented with chest and abdominal pain with peritoneal signs. His initial laboratory findings revealed leukocytosis with a WBC of 18,580/μL and a CRP of 0.2 mg/dL. A whole-body CT scan revealed a pulmonary contusion, multiple fractures (left clavicle, T12, L2, T4-6 vertebrae, left tibia, and fibula), and free air in the right upper quadrant (Figure 2a). A diagnostic laparoscopy was performed, which revealed no signs of hemorrhage, contamination, or visceral perforation (Figure 2b). Given the severe crushing mechanism, an abdominal drain was placed in the pouch of Douglas to monitor for any occult injury. The abdominal drain was removed on the second postoperative day. The patient was transferred to another hospital on the fifth postoperative day for the surgical management of his orthopedic injuries.

Case 3

**Figure 3:**
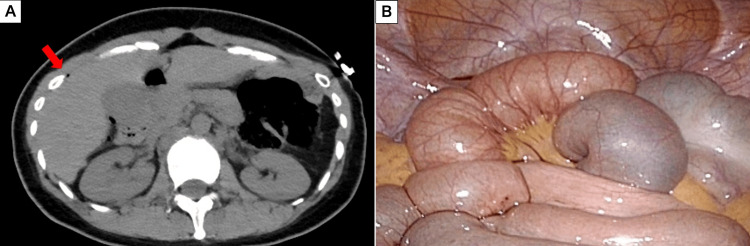
Imaging and intraoperative findings of Case 3. (A) Axial CT image demonstrating a small amount of free gas on the ventral side of the liver (red arrow). (B) A comprehensive laparoscopic exploration of the entire abdominal cavity revealing no evidence of hollow viscus perforation or contamination.

A 37-year-old female with a history of schizophrenia was transported to our hospital after jumping from the fifth floor of a building and landing on the third floor. On arrival, she was hemodynamically stable with a blood pressure of 115/70 mmHg and a heart rate of 80 beats/minute. Her abdomen was unremarkable on initial examination. Laboratory tests showed a WBC of 7,600/μL and CRP of 0.6 mg/dL. A whole-body CT scan performed immediately upon arrival revealed multiple injuries, including a right pneumothorax, pneumomediastinum, and thoracolumbar and sacral compression fractures. The scan also identified a small amount of free air around the liver along with the thoracic injuries (Figure 3a). A single-incision diagnostic laparoscopy was performed, which confirmed the absence of any digestive fluid leakage or perforation (Figure 3b). Given the high-energy mechanism of injury, an abdominal drain was placed in the pouch of Douglas as a precautionary measure. The patient had a smooth postoperative recovery. The abdominal drain was removed on the second postoperative day, and she was transferred to a psychiatric hospital on the 15th hospital day for continued care.

The clinical details and associated injuries of the three patients are summarized in Table 1.

**Table 1:** Summary of cases. ISS: Injury Severity Score; POD: postoperative day

	Case 1	Case 2	Case 3
Patient	48-year-old female	27-year-old male	37-year-old female
Psychiatric history	Schizophrenia	None	Schizophrenia
Mechanism of injury	Fall from the fifth floor, landed on a car	Crushing injury (700-kg panel)	Fall from the fifth to third floor
Associated injuries	Traumatic subarachnoid hemorrhage, bilateral hemopneumothorax, multiple rib, pelvic, and thoracolumbar fractures	Pulmonary contusion and multiple fractures (clavicle, spine, tibia/fibula)	Right pneumothorax, pneumomediastinum, and thoracolumbar and sacral compression fractures
ISS	34	8	17
Surgical approach	Diagnostic laparoscopy + mini-laparotomy	Diagnostic laparoscopy (three-port)	Single-incision diagnostic laparoscopy
Intraoperative findings	No perforation and retroperitoneal hematoma	No perforation	No perforation
Outcome and disposition	Transferred to neurosurgery on POD 10	Transferred for orthopedic surgery on POD 5	Transferred to a psychiatric hospital on POD 15

## Discussion

The management of traumatic pneumoperitoneum presents a significant clinical dilemma. While free intraperitoneal air is traditionally considered a hallmark of hollow viscus perforation, the existence of non-surgical causes is also well-documented in the literature [1,2]. Such non-perforating etiologies are reported to account for approximately 10% of all pneumoperitoneum cases [1]. Our case series clearly illustrates this challenge in the trauma setting, where high-energy mechanisms of injury further increase the suspicion of visceral perforation.

This diagnostic uncertainty is heightened by studies concluding that pneumoperitoneum on CT can be an unreliable predictor of intra-abdominal injury in blunt trauma [3]. Indeed, the prevalence of this challenge is significant; in a key study by Marek et al., the rate of negative laparotomy among blunt trauma patients with pneumoperitoneum was 21% [3]. This creates a difficult decision-making process for surgeons, who must weigh the risk of a negative laparotomy against the risk of a delayed diagnosis. This challenge is further illustrated by case reports of negative laparotomies following high-energy motor vehicle accidents [5], crushing injuries [6], and falls [7,8].

A key mechanism for non-surgical pneumoperitoneum in trauma is the influx of air from the thoracic cavity. Severe blunt chest trauma can cause alveolar rupture, leading to pneumomediastinum and/or pneumothorax [9]. This trapped air can then travel down through natural openings in the diaphragm, such as the esophageal hiatus, and enter the abdominal cavity. This thoracic origin mechanism provides a plausible explanation for the free air observed in our patients. This is strongly suggested by the specific thoracic injuries present: bilateral hemopneumothorax in Case 1, and a right pneumothorax with pneumomediastinum in Case 3. Pneumomediastinum, in particular, is a known cause of non-surgical pneumoperitoneum, as air can dissect along fascial planes from the chest into the abdomen [8,9].

In our three cases, the decision to perform diagnostic laparoscopy was made in hemodynamically stable patients to definitively exclude a visceral injury. The educational value of our series lies in demonstrating a practical clinical pathway: when faced with the dilemma of pneumoperitoneum in a stable, multi-trauma patient, diagnostic laparoscopy can serve as a definitive step to avoid an unnecessary and more morbid open exploratory laparotomy. This approach is consistent with other case reports where laparoscopy was instrumental in confirming the absence of perforation and preventing unnecessary surgery [10,11].

Limitations

This study has several limitations. As a retrospective case series with a small sample size, the findings cannot be generalized. There was no control group of patients managed with laparotomy or non-operative observation. Furthermore, the proposed thoracic origin of the pneumoperitoneum remains a plausible explanation rather than a definitively proven cause. However, despite these limitations, this series provides valuable insights into the clinical decision-making process and reinforces the educational message that traumatic pneumoperitoneum does not always equate to visceral perforation.

## Conclusions

In our series, diagnostic laparoscopy proved to be a useful tool to navigate the diagnostic uncertainty of traumatic pneumoperitoneum. By confirming the absence of visceral injury, we avoided negative laparotomies in these patients. Our experience suggests that for hemodynamically stable trauma patients with pneumoperitoneum, particularly when a thoracic injury is also present, diagnostic laparoscopy is a valuable procedure to consider for ensuring patient safety and preventing unnecessary surgical morbidity.
